# Predictors of stockpiling behavior during the COVID-19 pandemic in Germany

**DOI:** 10.1007/s10389-022-01727-x

**Published:** 2022-07-27

**Authors:** Kathrin Sadus, Jan Göttmann, Anna-Lena Schubert

**Affiliations:** 1 Institute of Psychology, Ruprecht Karl University Heidelberg, Hauptstraße 47–51, 69117 Heidelberg, Germany; 2 Department of Psychology, Johannes Gutenberg University Mainz, Wallstraße 3, 55122 Mainz, Germany

**Keywords:** COVID-19, Stockpiling, Health belief model, Social norms

## Abstract

**Aim:**

With the COVID-19 pandemic, we witnessed an increase in purchases of certain products, such as toilet paper, disinfectants, or groceries. In the present study, we examined the individual and socio-psychological determinants of stockpiling behavior. For this purpose, we defined an explanatory model based on the Health Belief Model (HBM), which includes threat perceptions, barriers and benefits, and self-efficacy beliefs as main predictors of health-related behaviors, and extended the model to include social norms.

**Subject and methods:**

Participants were recruited via social media platforms and data collection was conducted via an online survey. The final sample included 861 German respondents (male = 199, female = 642, mean age = 36.76, SD = 12.38).

**Results:**

Perceived barriers of stockpiling, such as financial constraints or regulations in supermarkets, turned out to be the strongest predictors of stockpiling. Regarding the role of threat perception, the perceived severity of the disease in particular was positively related to stockpiling behavior. Finally, our results suggest a significant impact of social cues, showing that descriptive normative beliefs are associated with stockpiling behavior.

**Conclusion:**

Based on these findings, we propose targeted interventions to a) reduce perceived benefits of stockpiling and severity beliefs related to COVID-19, b) emphasize disadvantages of stockpiling, and c) reduce media exposure of stockpiling behavior to prevent panic buying.

The coronavirus (COVID-19) outbreak evolved into a worldwide health crisis bearing severe social and economic disruptions. By January 30, 2020, the World Health Organization had declared a global emergency (Sohrabi et al. 2020), and on March 11, 2020, COVID-19 was officially proclaimed a pandemic. The progression of the disease triggered a substantial increase in the purchase of various products such as pasta, flour, and yeast, but most notably toilet paper (Statistisches Bundesamt 2020). In a survey published by the Institute for Interdisciplinary Research on Conflict and Violence at the University of Bielefeld, more than half of over 3000 German respondents stated they had stockpiled (Rees et al. 2020). While rational purchases are aimed at meeting daily needs, stockpiling is characterized by purchases far in excess of typical requirements. Such excessive purchasing has been frequently reported during humanitarian crises in various regions around the world (Yuen et al. 2020), but the stockpiling behavior in the wake of the COVID-19 pandemic in particular triggered investigations on the underlying psychological factors. Understanding the mechanisms is important, as the consequences of stockpiling behavior are severe and could ultimately result in people who need them most not being able to purchase essential goods, and in fluctuating prices for important products such as masks or disinfectants (Chen et al. 2020).

This raises the question as to what differentiates people who started stockpiling from those who continued with their regular shopping behavior. Several studies have investigated the psychological determinants of stockpiling in the course of the COVID-19 pandemic, mainly focusing on personality as an explanatory variable. Studies consistently show a significant relationship between honesty–humility and stockpiling, suggesting that people who do not seek personal gain and follow fair rules are less likely to stockpile (Columbus 2021; Fischer et al. 2021; Rudert and Janke 2021), with only one study finding no significant relationship between honesty–humility and stockpiling (Garbe et al. 2020). Results on the relationship between other personality dimensions and hoarding are somewhat more mixed. While some researchers report that higher emotionality is associated with more stockpiling (Dammeyer 2020; Fischer et al. 2021; Yoshino et al. 2021), other studies find no evidence of such a relationship (Garbe et al. 2020; Zettler et al. 2022). The findings are similar with regard to the influence of conscientiousness. While some studies report no effect (Yoshino et al. 2021; Zettler et al. 2022), there is evidence for a negative association between conscientiousness with over-purchase (Dammeyer 2020) and with hoarding face masks and hand sanitizers (Aschwanden et al. 2020), while Garbe et al. (2020) report a positive association between hoarding toilet paper and conscientiousness.

When examining other predictors of stockpiling, several studies consistently found that individuals who felt more threatened by COVID-19 reported higher intention to stockpile (Giroux et al. 2021; Kim et al. 2020) and were more likely to build up stocks (Garbe et al. 2020; Nowak et al. 2020). However, most studies focused on the threat of COVID-19 and did not consider the threat of being unable to purchase essential items due to shortages, which can have a direct impact on the need to stockpile. In a first study, Lehberger et al. (2021) for instance found a significant relationship between stockpiling intention and fear of future unavailability.

Taken together, studies consistently reported effects of honesty–humility and threat perceptions as main drivers of stockpiling behavior. In addition, there is also evidence for an influence of descriptive normative information, in the sense that people who rate the prevalence of stockpiling behavior in their environment as higher also tend to build up stocks (Columbus 2021; Lehberger et al. 2021; Rudert and Janke 2021).

The present study constitutes a comprehensive investigation of potential factors influencing stockpiling behavior and includes the examination of personality traits and individual threat beliefs as well as influences of normative social cues.^1^ To this end, we developed a framework based on the Health Belief Model (HBM) (Kirscht 1974; Rosenstock et al. 1988; Strecher and Rosenstock 1997), which places a strong emphasis on threat beliefs, and extended it to include other concepts that have been shown to be important predictors in explaining behavior (see Fig. 1).

**Fig. 1:**
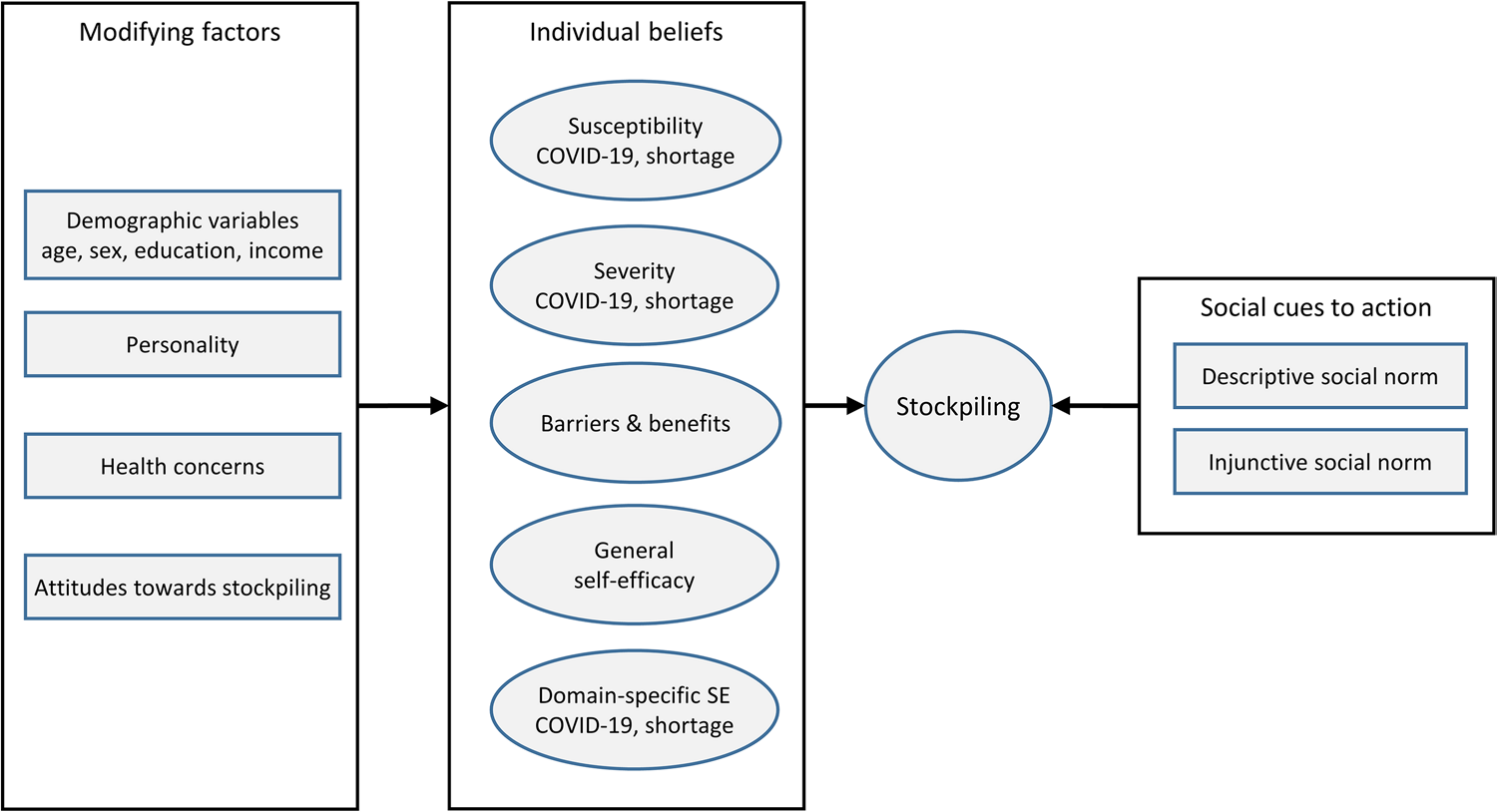
Conceptualization of determinants of stockpiling. *SE* = self-efficacy

## Explanatory model of stockpiling

The HBM has traditionally been used to investigate a broad range of health-related behaviors, such as smoking (Sharifi-rad et al. 2007) and screening behavior (Yarbrough and Braden 2001). The HBM postulates that decision-making regarding health-related behaviors depends on individual beliefs which include barriers and benefits of health-promoting behaviors as well as threat perceptions that are categorized into perceived susceptibility and severity. The HBM further states that modifying factors such as personality traits or demographic variables directly influence the respective beliefs (Champion and Skinner 2008).

Together, these constructs have been used to predict under what circumstances people perform or change behaviors, even some of those that fall outside the scope of health behaviors but are likewise related to perceptions of vulnerability and severity of negative consequences, such as gambling (Tong et al. 2019) or recycling (Lindsay and Strathman 1997). Similarly, stockpiling has substantial resemblances to health-related behaviors; it serves to maintain well-being and quality of life and is related to perceived threats to well-being. One difference from health behavior, however, is the extent to which stockpiling involves trade-offs with the welfare of others and thus also presents a social dilemma (Van Lange et al. 2013), where hoarding is tempting in that it leads to better short-term outcomes for individuals. However, if everyone starts stockpiling, it leads to more negative consequences in the longer term (e.g., high price fluctuation, unavailability of essential products) than if everyone had followed their normal shopping routine. In many ways, stockpiling can be conceived of as a “tragedy of the commons” (Hardin 1968), where shared resources are depleted because individuals act mostly in their self-interests.

Nonetheless, we suggest that the HBM is particularly suitable as a baseline model, as it attributes a central and differentiated role to threat perceptions that were associated with stockpiling in previous studies (Garbe et al. 2020; Kim et al. 2020) and also allows for the integration of other relevant predictors such as personality factors or social norms.

### Perceived susceptibility

Perceived susceptibility refers to beliefs regarding the likelihood of experiencing an undesirable condition. Several meta-analyses found small but consistent effects of susceptibility beliefs on behavior (Carpenter 2010; Harrison et al. 1992). Considering the high media coverage of COVID-19 infection rates and the constant warnings of the risk of infection, it seems plausible that vulnerability perceptions during the pandemic have had a major impact on behavior. Thus, in the scope of the present study, we investigated how the perceived susceptibility to suffering from COVID-19 is related to stockpiling. Considering the basic motivation for stockpiling to avoid a shortage of essential products, we investigated not only disease-related perceptions of susceptibility but also included the perceived vulnerability to experiencing shortages. Recent research indeed suggests that people who are threatened by the potential shortage of indispensable products are more prone to stockpile (Lehberger et al. 2021).

### Perceived severity

According to the HBM, behavior can be also explained by the severity attributed to particular conditions. The model predicts that the more people fear an impairment of their living conditions, the more likely they avoid undesirable health consequences and take preventive measures. In an early published meta-analysis, the effect of perceived severity was particularly important for behaviors that aim at risk reduction (e.g., screenings) compared to other behaviors (Harrison et al. 1992). For this reason, perceived severity is particularly relevant, since hoarding can be understood as a measure for risk reduction. Giroux et al. (2021) showed that providing information on the prevalence and mortality associated with COVID-19, compared with prevalence alone and thus emphasizing the severity, increased stockpiling intention. In a study by Nowak et al. (2020) who also investigated stockpiling behavior based on the HBM, it was shown that both perceived severity and perceived vulnerability could predict hoarding. In the present study, we assessed the perceived severity of suffering from COVID-19, assuming that the more severe people perceive COVID-19 to be (e.g., because they expected far-reaching consequences for themselves and their loved ones), the more likely they are to hoard. An additional question arises as to the role of impending scarcity in predicting stockpiling behavior beyond the perceived threat posed by COVID-19. Thus, we expected that consumer behavior would also depend on the perceived severity of a possible shortage, i.e., that people would be more inclined to hoard if they regarded it as particularly serious not to have products available in retail stores.

### Perceived barriers and benefits

Within the HBM, the likelihood of taking certain actions also depends on perceived benefits and barriers. While perceived benefits are derived from the potential effectiveness of particular actions in reducing the threat that emanates from the disease/illness, perceived barriers constitute opposing constraints such as inconvenience, time, or foregoing something positive. In a meta-analytic synthesis, benefits and barriers were the strongest predictors of health-related behaviors (Carpenter 2010). Related to the present study, these constructs suggest a cost–benefit analysis of panic buying, resulting in a direct influence on the probability of hoarding. Interestingly, in the context of stockpiling, Nowak et al. (2020) found perceived barriers of preventive measures (e.g., not having enough time to apply preventive measures) to be a significant predictor of stockpiling intention and behavior, while perceived benefits like frequently washing hands were not related to an increased purchasing behavior. Roșu et al. (2021), on the other hand, examined the influence of barriers to and benefits of refraining from stockpiling and adjusting consumption back to sustainable levels, assuming that people were engaged in stockpiling. They found that higher perceived barriers (e.g., adjusting current consumption to actual needs required effort) were associated with more stockpiling intentions and behavior, while benefits were not. Therefore, the question remains open whether the effects of perceived benefits can also be generalized in the context of stockpiling.

### General self-efficacy

Although the original HBM did not include self-efficacy, it was added to the core components in later revisions (Rosenstock et al. 1988). Self-efficacy describes the belief in one’s own ability to perform the actions required to achieve certain goals (Bandura 2010). Individuals with high self-efficacy are more confident that they can overcome problems and adapt to changing and challenging conditions, thus affecting their risk perception and coping behavior (Bandura 2012; Witte 1992).

### Domain-specific self-efficacy concerning COVID-19 and shortages

Despite the undisputed relevance of general self-efficacy, research emphasizes the importance of domain-specific beliefs about the ability to perform a task for the prediction of behavior, suggesting that both kinds of beliefs, although correlated, still should be conceptualized as separate constructs (Grether et al. 2018). Consequently, we included a measure of general self-efficacy and two domain-specific control beliefs, which refer to a) the self-efficacy regarding the coping of health-threatening aspects of COVID-19 and b) the trust in one’s ability to manage possible shortages of commodities. We expected that people with higher levels of general and domain-specific self-efficacy were more likely to rely on their capability of mastering the COVID-19 pandemic and therefore less tempted to prepare themselves for the worst-case scenario. In line with the revision of the HBM, we included general and domain-specific self-efficacy as part of the core constructs.

### Modifying factors

Modifying factors are described as a diverse set of demographic and socio-psychological variables. Although these modifying factors are not interpreted as directly causal for shaping health-related behavior, it is assumed that they indirectly influence the probability of taking health-related measures by altering the perception of threats as well as the costs and benefits of these measures. As a personality measure, we included the HEXACO inventory (Garbe et al. 2020; Lee and Ashton 2008), which extends the Big 5 model of personality by the dimension honesty-humility.

### Further model extensions

Furthermore, we included attitudes towards stockpiling and social norms, two constructs that were useful predictors in other theoretical frameworks, most notably the theory of planned behavior (Ajzen 2011). As attitudes are commonly characterized on three dimensions, namely cognitive, affective, and behavioral (Rosenberg and Hovland 1960), we included cognitive and affective attitudes towards stockpiling. Furthermore, we also assessed health concerns, as these might directly modify individual threat beliefs regarding COVID-19. Finally, we included social norms, which provide important information about what behavior is common and socially acceptable and serve as principles by which we guide our behavior in social contexts. Cialdini et al. (1990) differentiated between perceived acceptability (injunctive norm) and observable performance of behaviors (descriptive norm). Thus, injunctive norms describe what is believed to be morally approved or disapproved by the majority, while descriptive norms refer to the perception of what is typically done by others.

While some researchers examined the role of descriptive norms finding that they predicted stockpiling behavior (Columbus 2021; Lehberger et al. 2021; Rudert and Janke 2021), the influence of injunctive norms is less studied. The inclusion of injunctive norms is an important extension, as stockpiling behavior is notoriously associated with negative attributes and depicted as shameful and selfish (Phillips et al. 2021). One study included injunctive social norms and found no relationship between injunctive normative information and stockpiling (Roșu et al. 2021). However, Roşu et al. (2021) conceptualized injunctive norms by asking whether significant others believe a product shortage is likely, which does not include a normative assessment of the acceptability of the behavior in question. Thus, a more specific examination of social norms is necessary to understand the nature of the relationship between norms and stockpiling.

The HBM conceptualizes such external factors as cues to action that directly influence the probability of taking action (Champion and Skinner 2008). Therefore, we used normative beliefs with regard to the commonness and acceptability of stockpiling as external cues to actions that predict stockpiling behavior.

## The present study

The present study aimed to investigate stockpiling behavior in a German sample using an explanatory framework based on the HBM which was extended to include relevant predictors identified by previous research on the prediction of behavior. Therefore, we used a multiple indicator assessment of stockpiling that included a wide range of product groups and employed a latent variable approach to ensure a comprehensive measurement of stockpiling behavior. We extend the results on the relationship between hoarding and threat perceptions, by including not only the perceived threat of COVID-19 but also the perceived threat of experiencing product shortages. In addition, as suggested by the HBM, we did not assess threat as a unidimensional construct, but distinguished between perceived severity and perceived susceptibility. Thus, we examined four aspects of potential threats: perceived severity and susceptibility to COVID-19 as well as perceived severity and susceptibility to a shortage.

Taken together, the study has three main objectives: 1) an integration of previous findings in the context of an explorative application of an HBM-based model, 2) a differentiated consideration of previous findings on the relationship between threat perceptions and stockpiling behavior by assessing threat perceptions related to the disease itself and the threat posed by the potential shortage of goods, and 3) a consideration of descriptive and injunctive normative information.

Based on our results, we aimed to advance the understanding of the mechanisms underlying stockpiling behavior to facilitate effective communication and develop target countermeasures against panic buying in the future.

## Method

The study materials, dataset, and analysis script are available on the Open Science Framework (OSF; https://osf.io/xyct7/).

### Participants

Out of *n* = 866 German participants who completed the online survey, *n* = 5 were excluded during the data preprocessing, resulting in a final sample of *n* = 861 (female = 642, male = 199, mean age = 36.76, SD = 12.38). This gave us a power of 1−ß = 1.00 to test the hypothesis of close fit (H0: ε ≤ .05, H1: ε ≥ .10) as suggested by Browne and Cudeck (1992). Participants were recruited on social media platforms between April 2nd and May 2nd, 2020. For further sample information see Table 1. Only persons 18 years of age or older were included. All participants gave their informed consent. Participants received no compensation.

**Table 1:** Sample demographics

	*N*	%
Sex		
Male	199	23.11
Female	642	74.56
No information	20	2.32
Education		
Elementary education	155	18.00
Secondary education	316	36.70
University degree	367	42.62
No information	23	2.67
Income^a^		
No own income	46	5.34
Low income	145	16.84
Middle income	408	47.39
High income	202	23.46
No information	60	6.97
Employment		
In training/student	171	19.86
Unemployed	25	2.90
Employed	540	62.72
Self-employed	55	6.39
No information	70	8.13

^*a*^based on a question for the net household income assessed with nine categories (low = less than 250–999 €, middle = 1000–2499 €, high = 2500 € and above)

## Materials

We employed an online survey to assess stockpiling behavior and the above-mentioned constructs. All items were measured with 5-point Likert rating scales ranging from 1 (very inaccurate) to 5 (very accurate) unless otherwise noted. The various constructs were surveyed in the order presented here, while the items were randomly presented within a scale.

### Stockpiling

To assess stockpiling, we presented a list of product groups. Participants were asked to think about the purchases they had made in the period since the COVID-19 pandemic and to indicate whether and to what extent their purchasing behavior had deviated from their regular purchases. Given that the time at which individuals realized that COVID-19 was a global health crisis varied, we did not set a specific time period. Instead, we opted for an instruction with a variable time period that incorporates this variability to provide a valid measure of COVID-related changes in shopping behavior. We used a 7-point Likert scale allowing participants to rate whether they bought more, the same, or less of the respective products (α = .77). Participants could also indicate that they generally did not buy certain product groups at all.

### Susceptibility and severity

Susceptibility to the lack of essential products was measured with five self-generated items (e.g., "I am very concerned that certain products will not be available in the near future"). Similarly, we used four self-generated items to assess the perceived susceptibility to a COVID-infection (e.g., "I estimate the probability that I will suffer from COVID to be high"). The severity of not being able to buy all products was measured using seven self-generated items (e.g., "If shopping in supermarkets is limited, that would affect me severely"). Finally, five self-generated items were administered to assess the perceived severity of an infection (e.g., "The idea of being sick with coronavirus scares me"). For all scales, internal consistency was acceptable with values ranging from α = .67 to .73 (see Appendix D).

### Barriers and benefits

Perceived benefits were assessed through three self-generated items (e.g., "If I have enough everyday products in stock, I feel safe"), *α* = .70. Perceived barriers were measured with four self-generated items, including the difficulty of making large purchases due to limited financial and transportation resources or corresponding governmental regulations. The internal consistency was low at α = .29, mainly due to the wide range of possible barriers.

### Self-efficacy

General self-efficacy was assessed with the German version of the General Self-Efficacy Scale (SWE; Schwarzer and Jerusalem 1995). This scale assesses general self-efficacy with ten items (e.g., "I always succeed in solving difficult problems if I make an effort") and has been validated in over 28 languages (Scholz et al. 2002). Respondents were asked to indicate whether they agreed with the statements on a scale ranging from 1 (not at all true) to 4 (exactly true), α = .87.

Domain-specific self-efficacy was assessed according to threat beliefs. Thus, we assessed the extent to which people felt capable to cope with a shortage of products (e.g., "If certain products are no longer available due to a shortage of supply, I am confident that a pragmatic solution can be found") and the perceived capability to cope with a COVID-19 infection (e.g., " If I had to go into domestic quarantine because of the coronavirus, I'm sure I could find good solutions to most of the problems associated with it"). The scales were measured with four self-generated items each, with all αs > .70.

### Modifying factors

We administered the HEXACO-60 personality inventory (Ashton and Lee 2009; Moshagen et al. 2014), which provides a reliable measure of six major dimensions of personality: honesty–humility, emotionality, extraversion, agreeableness, conscientiousness, and openness to experience with ten items for each domain-level scale. Participants were asked to indicate their agreement with each item on a scale ranging from 1 (strongly disagree) to 5 (strongly agree). Previous research provides evidence of the reliability and validity of the HEXACO-60 (Moshagen et al. 2019). The internal consistency was acceptable, with all αs >.70.

To measure attitudes towards stockpiling, we presented semantic differential scales as response format (e.g., "reasonable vs unreasonable", "reassuring vs disquieting") and used three items each to measure affective attitudes, α = .68, and cognitive attitudes, α = .84, respectively. In addition, we used the modified version of the Short Health Anxiety Inventory (mSHAI) (Salkovskis et al. 2002) to measure health concerns, α = .94.

### Social norms

Descriptive and injunctive social norms were assessed through three self-generated items each. Participants were asked to indicate the perceived prevalence of people in their environment who engaged in panic buying on a scale from 0 to 100 percent in 10-percent steps, α = .70 (e.g., "How many of the people around you bought more products during the Corona epidemic compared to their purchases before the epidemic?"). Items measuring the injunctive social norm were framed as statements about the perceived acceptability of stockpiling. Participants were asked to indicate their agreement with each of the three statements on a 7-point Likert scale ranging from 1 (fully disagree) to 7 (fully agree), α = .70. (e.g., "If I buy some products during the Corona epidemic in larger quantities than would be usual for me, most people around me would approve of this").

## Data analysis

Five subjects were removed because they reported being under 18 years old and thus did not meet the inclusion criterion. We used the maximum likelihood estimation with robust standard errors to account for violations of the normality assumption. Product groups rated as "I don’t buy at all" were coded as missings so that they were not included in the weighting of stockpiling. We used the statistics software R – version 4.1.0 (R. Core Team, 2021) for data preprocessing and analyses.

The following packages were used in R: for preparation and data-management we used the package “dplyr” version 1.0.8 (Wickham et al. 2022) and the package “MVN” version 5.9 (Korkmaz et al. 2014), for descriptive statistics the package “psych” version 2.2.3 (Revelle 2022), for structural equation model analyses the package “lavaan” version 0.6-11 (Rosseel 2012) and the package “semTools” version 0.5-5 (Jorgensen et al. 2021), and for visualizations the package “ggcorplot” version 0.1.3 (Kassambara 2019).

## Structural equation modeling

We defined a measurement model with a latent variable for stockpiling behavior, indicated by the purchase ratings of all products. To predict stockpiling behavior according to the theoretical assumptions of our model, we defined a structural model which predicts stockpiling behavior through the core constructs (perceived susceptibility and severity, benefits and barriers, general and domain-specific self-efficacy). These variables were in turn predicted by the modifying factors (demographic variables, personality, health concerns). For the prediction of general and domain-specific self-efficacy, we included affective and cognitive attitudes, as the literature suggests a close relationship between self-efficacy and attitudes (De Vries et al. 1988; Topa and Moriano 2010). Since cues to action are expected to have a direct effect on behavior in the HBM, we defined a direct path from social normative cues to stockpiling behavior. To assess the model fit, we refer to the conventional cut-off criteria proposed by Hu and Bentler (1999) who recommend a cutoff value of .08 for SRMR, a cutoff value of .06 for RMSEA, and a cutoff value of .95 for the CFI, to conclude that the hypothesized model provides a good fit to the data.

## Results

The correlations between all constructs of interest are displayed in Appendix A Table 8.

### Structural equation modeling

To fit the structural model, we first analyzed the factor structure of stockpiling and validated the measurement model. Therefore, we first report the fit statistics of the measurement model and then proceed to the results of the path model.

### Measurement model of stockpiling

To analyze the factor structure of stockpiling, we conducted a principal components analysis. The inspection of the respective scree plot revealed a clear one-factorial solution supporting the unidimensionality of stockpiling (see Appendix B). Accordingly, we defined a measurement model including one latent factor.

Model fit of the measurement model was good (χ^2^ (209) = 465.25; RMSEA = .045 [.040 .051], SRMR = .07), with one exception (CFI = .79). However, the CFI is not interpretable if the RMSEA of the baseline model is lower than .158 (Kenny 2020). For the present model, the baseline RMSEA is .09, which implies that the CFI is not informative (Table 2).

**Table 2:** Mean and standardized factor loadings of all product groups assessed

Products	*M*	*SD*	β	[95% boundaries]	*p*
Dairy products	4.25	0.64	.45	[ .34; .56]	< .001
Sweetener	3.89	0.54	.38	[ .16; .60]	.001
Convenience and frozen products	4.18	0.82	.46	[ .38; .54]	< .001
Grain-products and pasta (noodles, rice etc.)	4.33	0.71	.58	[ .51; .66]	< .001
Meat, fish and eggs (fresh)	4.15	0.62	.27	[ .16; .37]	< .001
Baked goods (not deep-frozen)	4.08	0.73	.27	[ .18; .36]	< .001
Sweets and snacks	4.34	0.88	.27	[ .17; .38]	< .001
Sauces and dips	3.93	0.59	.38	[ .23; .54]	< .001
Spices and oils	4.02	0.48	.47	[ .34; .60]	< .001
Toilet paper	3.99	0.85	.49	[ .40; .57]	< .001
Contraceptives	3.98	0.50	.22	[ .04; .41]	.039
Cosmetics and hair care products	3.89	0.65	.39	[ .24; .55]	< .001
Cleaning agents, disinfectants and detergents	4.19	0.66	.47	[ .38; .57]	< .001
Kitchen utensils	3.97	0.52	.49	[ .38; .61]	< .001
Canned food	4.30	0.81	.54	[ .45; .64]	< .001
Fruit and vegetables (fresh)	4.36	0.73	.13	[ .05; .21]	.001
Alcoholic beverages	4.05	0.95	.18	[ .05; .32]	.007
Non-alcoholic beverages	4.28	0.67	.32	[ .19; .44]	< .001
Tobacco products	4.17	0.90	.13	[-.03; .28]	.104
Medication	4.03	0.64	.39	[ .28; .50]	< .001
Pet food	4.32	0.66	.47	[ .32; .61]	< .001
Baby products	4.32	0.64	.40	[ .18; .61]	.001

χ^2^ (209) **=** 465.25; RMSEA = .045 [.040 .051], SRMR = .07, CFI = .79

Exactly those product groups turned out to be particularly good indicators of stockpiling that have been consistently associated with panic buying, such as canned food, grain products, toilet paper, and cleaning agents.

## Path model

Due to the complexity of the path model, we first report on the relationship between stockpiling and individual beliefs (susceptibility, severity, barriers, benefits, and self-efficacy) and then move on to the predictors of these beliefs. Although the results will be presented separately, all structural relationships were modeled within the same model. The model provided a good fit to the data, χ2 (2141) = 5998.04, RMSEA = .049 [.047 .050], SRMR = .07, except for the CFI = .73. Again, the RMSEA of the baseline model was lower than the recommended benchmark of .158 (RMSEA_Baseline_ = .09), indicating that the CFI was not an appropriate measure of model fit.

### Stockpiling

**Fig. 2:**
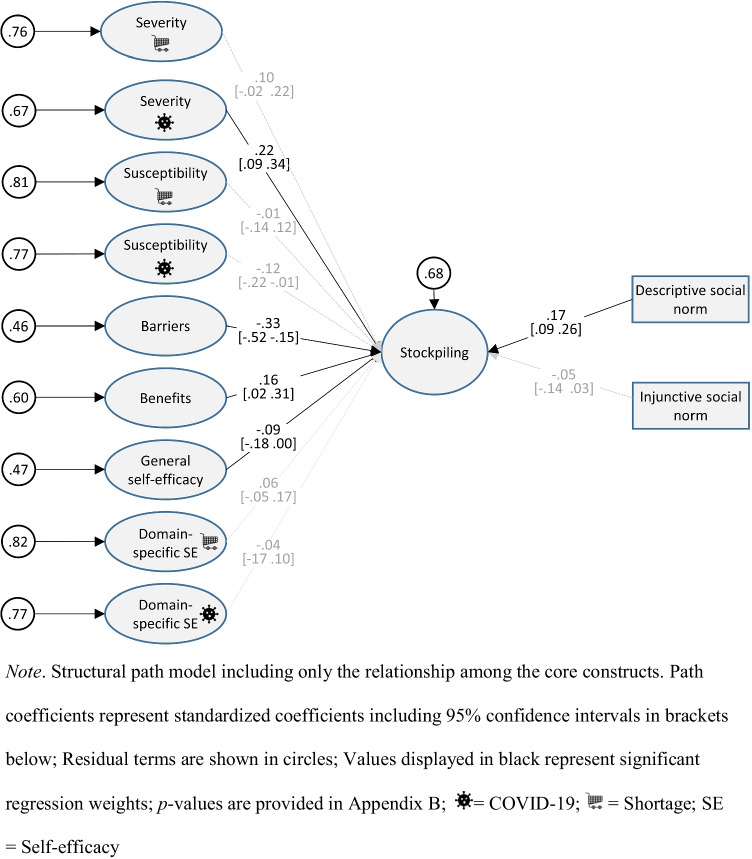
Relationship among latent factors in the path model. *Note*. Structural path model including only the relationship among the core constructs. Path coefficients represent standardized coefficients including 95% confidence intervals in *brackets below*; Residual terms are shown in *circles*; Values displayed in *black* represent significant regression weights; *p*-values are provided in Appendix C;  = COVID-19;  = shortage; SE = self-efficacy

As displayed in Fig. 2, perceived barriers (β = *−.*33) and benefits (β = *.*16) were associated with stockpiling. Among threat perceptions, only the perceived severity of a COVID-19 infection was a significant predictor of stockpiling (β = *.*22), suggesting that panic buying was not motivated by the actual fear of a product shortage, but by the fear of possible infection.

Moreover, descriptive normative beliefs were related to stockpiling (β = *.*17), while injunctive norms failed to predict stockpiling behavior (β = *−.*05), suggesting that observing actual behavior showed a stronger relationship with stockpiling than perceiving what is morally acceptable. Finally, general self-efficacy also proved to be a significant but weaker predictor of stockpiling (β = -.09).

### Severity and susceptibility

The perceived severity of a COVID-19 infection was related to health concerns (β = .41) and age (β = .21), indicating that elderly people and people who were more concerned about their health in general perceived the consequences of possible infection as more severe than younger and less concerned people. Similarly, health concerns (β = .36) and age (β = .29) were significant predictors of perceived susceptibility to suffering from an infection. Among the personality dimensions, only emotionality turned out to be a significant predictor of perceived severity of infection (β = .22) (Table 3)

**Table 3:** Predictors of perceived susceptibility and severity of COVID-19 infection

Variables	β	[95% CI]	*p*	β	[95% CI]	*p*
	Severity *→*			Susceptibility *→*		
Extraversion	-.05	[-.13; .03]	.195	-.06	[-.13; .01]	.098
Emotionality	.22	[ .14; .30]	< .001^***^	.02	[-.06; .09]	.645
Openness	-.01	[-.08; .06]	.844	.05	[-.02; .12]	.204
Agreeableness	.01	[-.06; .08]	.737	-.02	[-.09; .05]	.634
Conscientiousness	.03	[-.04; .09]	.471	.01	[-.05; .08]	.722
Honesty–humility	-.04	[-.12; .03]	.249	-.004	[-.07; .07]	.911
Health concerns	.41	[ .34; .49]	< .001^***^	.36	[.28; .44]	< .001^***^
Age	.21	[ .13; .30]	< .001^***^	.29	[.21; .38]	< .001^***^
Sex	-.02	[-.09; .05]	.520	.03	[-.04; .10]	.358
Education	.01	[-.07; .08]	.899	-.03	[-.11; .04]	.377
Income	-.06	[-.14; .01]	.113	-.01	[-.08; .06]	.780

* *p* < .05; ** *p* < .01; *** *p* < .001

In addition to the threat posed by infection, we investigated which factors contributed to the perception of the threat caused by a shortage of products in supermarkets. Although neither the susceptibility to a shortage nor the severity attributed to it turned out to be significant predictors of stockpiling, we briefly report the predictors of the respective beliefs to gain a better understanding of their relevance in predicting threat perceptions. Again, health concerns predicted the susceptibility to a shortage (β = .19) and how serious it was perceived (β = *.*14). However, the results revealed a slightly different pattern regarding the effects of personality traits, as compared to threat perceptions regarding a COVID-19 contraction. Both perceived severity and perceived susceptibility to a shortage of goods were associated with higher emotionality and lower extraversion, agreeableness, and honesty–humility. In addition, individuals who rated a shortage as more severe tended to report higher levels of openness, while participants who felt more vulnerable exhibited higher contentiousness (Table 4).

**Table 4:** Predictors of perceived severity to and susceptibility of a shortage

Variables	β	[95% CI]	*p*	β	[95% CI]	*p*
	Severity *→*			Susceptibility *→*		
Extraversion	-.11	[-.19; -.04]	*.*003^∗∗^	-.13	[-.21; -.04]	*.*003^∗∗^
Emotionality	.22	[.14; .30 ]	*<. *001^∗∗∗^	.13	[.03; .22]	*.*009^∗∗^
Openness	-.10	[-.17; -.02]	.010^∗^	.01	[-.07; .09]	*.*759
Agreeableness	-.08	[-.15; .003]	*.*043^∗^	-.12	[-.20; -.04]	*.*006^∗∗^
Conscientiousness	.06	[-.01; .13 ]	*.*093	.12	[ .04; .20]	*.*003^∗∗^
Honesty–humility	-.23	[-.31; -.16]	*< .*001^∗∗∗^	-.15	[-.24; -.05]	*.*002^∗∗^
Health concerns	.14	[.05; .23 ]	*.*002^∗∗^	.19	[ .09; .28]	*< .*001^∗∗∗^
Age	.05	[-.03; .13 ]	*.*235	.06	[-.03; .15]	.212
Sex	-.04	[-.10; .03 ]	*.*316	-.004	[-.09; .08]	*.*927
Education	-.03	[-.11; .04 ]	*.*373	-.10	[-.18; -.02]	*.*015
Income	-.01	[-.08; .07 ]	*.*840	-.02	[-.09; .06]	*.*711

* *p* < .05; ** *p* < .01; *** *p* < .001

### Barriers and benefits

Perceived barriers and benefits were both predicted mainly by cognitive attitudes towards stockpiling. Individuals who reported negative cognitive attitudes toward stockpiling, deeming it unreasonable and harmful, stated perceiving fewer benefits (β = *−.*46) and, above all, more barriers (β = .54). Interestingly, affective attitudes were not significantly related to perceived barriers (β = .10) but significantly associated with perceived benefits (β = −.13).

Moreover, honesty–humility was related to both benefits (β = −.15) and barriers (β = .16), such that higher scores were associated with fewer perceived benefits and more perceived barriers. Perceived benefits were also related to conscientiousness (β = .16). Finally, more extraverted participants also tended to report fewer benefits of stockpiling (β = *−.*11) than less extraverted ones. Among the demographic variables, the results imply that people with a higher income reported fewer barriers (β = −.22) than those with lower incomes, which might be due to the fact that we included financial constraints as a possible barrier to building up stocks (Table 5).

**Table 5:** Predictors of perceived barriers and benefits associated with stockpiling

Variables	β	[95% CI]	*p*	β	[95% CI]	*p*
	Barriers *→*			Benefits *→*		
Extraversion	-.08	[-.18; .02]	.117	-.11	[-.19; -.04]	.002^**^
Emotionality	.10	[-.01; .20]	.062	.06	[-.02; .14]	.133
Openness	-.002	[-.10; .09]	.971	.06	[-.01; .13]	.103
Agreeableness	.02	[-.08; .12]	.690	.06	[-.02; .14]	.145
Conscientiousness	-.03	[-.12; .06]	.523	.16	[ .09; .24]	< .001^***^
Honesty–humility	.16	[ .06; .26]	.004^**^	-.15	[-.24; -.07]	< .001^***^
Health concerns	-.07	[-.17; .04]	.191	.05	[-.04; .13]	.297
Affective attitudes	.10	[-.03; .22]	.153	-.13	[-.22; -.04]	.005^**^
Cognitive attitudes	.54	[ .41; .68]	< .001^***^	-.46	[-.56; -.36]	< .001^***^
Age	-.09	[-.22; .03]	.147	.08	[-.01; .17]	.086
Sex	-.04	[-.15; .06]	.407	.02	[-.06; .10]	.625
Education	-.03	[-.15; .09]	.623	-.03	[-.10; .05]	.494
Income	-.22	[-.35; -.10]	.005^**^	.02	[-.06; .11]	.613

* *p* < .05; ** *p* < .01; *** *p* < .001

### General self-efficacy

Among the personality dimensions, extraversion (β = *.*47) and conscientiousness (β = *.*18) turned out to be positively related to general self-efficacy, while emotionality was negatively associated with self-efficacy beliefs (β = *−.*41). At the same time, we found small effects of age (β = *−.*06), sex (β = *−.*10), and income (β = *.*09) (Table 6).

**Table 6:** Predictors of general self-efficacy

Variables	β	[95% CI]	*p*
General self-efficacy *→*			
Extraversion	.47	[ .41; .52]	< .001^***^
Emotionality	-.41	[-.47; -.34]	< .001^***^
Openness	.04	[-.02; .10]	.173
Agreeableness	-.003	[-.06; .06]	.924
Conscientiousness	.18	[ .12; .24]	< .001^***^
Honesty-Humility	-.01	[-.07; .05]	.711
Health concerns	-.03	[-.09; .04]	.471
Age	-.06	[-.13; .000]	.049^*^
Sex	-.10	[-.16; -.04]	.001^**^
Education	-.06	[-.12; .002]	.059
Income	.09	[ .03; .16]	.004^**^

* *p* < .05; ** *p* < .01; *** *p* < .001

We also included domain-specific self-efficacy beliefs, which assessed the trust in one’s abilities to deal with COVID-19 infection on the one hand and a potential shortage on the other hand. Self-efficacy regarding the coping abilities with illness was positively related to extraversion (β = *.*13). In contrast, emotionality (β = *−.*21), health concerns (β = *−.*24) and age (β = *−.*17) were negatively associated with infection-related self-efficacy. We also found extraversion (β = *.*13), openness (β = *.*13), agreeableness (β = *.*11) and honesty (β = *.*14) to be positively related to the confidence in one’s own capacity to cope with a shortage. Again, emotionality (β = *−.*18) and health concerns (β = *−.*11) showed the opposite relationship (Table 7).

**Table 7:** Predictors of domain-specific self-efficacy

Variables	β	[95% CI]	*p*	β	[95% CI]	*p*
	Specific self-efficacy *→*			Specific self-efficacy *→*		
Extraversion	.13	[.05; .21]	.001^**^	.13	[ .05; .20]	.002^**^
Emotionality	-.21	[-.30; -.13]	< .001^***^	-.18	[-.27; -.09]	<.001^***^
Openness	.05	[-.03; .12]	.245	.13	[ .05; .21]	.002^**^
Agreeableness	.05	[-.03; .12]	.198	.11	[ .04; .19]	.004^**^
Conscientiousness	.06	[-.01; .13]	.113	-.05	[-.13; .03]	.196
Honesty–humility	.02	[-.06; .10]	.618	.14	[ .06; .23]	.001^**^
Health concerns	-.24	[-.34; -.15]	< .001^***^	-.11	[-.20; -.02]	.024^*^
Age	-.17	[-.25; -.08]	< 001^***^	-.09	[-.17; .004]	.063
Sex	-.05	[-.12; .03]	.206	.02	[-.06; .09]	.687
Education	.02	[-.06; .09]	.619	.03	[-.05; .11]	.418
Income	.07	[-.01; .15]	.076	.02	[-.07; .10]	.721

* *p* < .05; ** *p* < .01; *** *p* < .001;  = COVID-19;  = shortage

## Discussion

The aim of the present study was to identify individual and social factors contributing to stockpiling behavior during the COVID-19 pandemic in Germany. Several exploratory attempts to explain stockpiling at the onset and during the pandemic progression identified relevant factors, such as personality traits (Aschwanden et al. 2020; Columbus 2021; Dammeyer 2020; Yoshino et al. 2021; Zettler et al. 2022) and threat perceptions (Garbe et al. 2020; Giroux et al. 2021; Kim et al. 2020; Nowak et al. 2020). In the present study, we defined an explanatory framework to understand the relationships between stockpiling and social and psychological variables during the early stages of the COVID-19 pandemic. The particular strength of this study is the multi-indicator measurement of stockpiling and the comprehensive analysis of demographic, psychological, and social factors. With regard to the main goals of our study, our results indicate that threat perceptions and descriptive social norms were relevant personal beliefs for the prediction of panic buying. By including constructs from the HBM, we were able to demonstrate that perceived benefits and barriers were also associated with stockpiling behavior.

Consistent with evidence suggesting that hoarding of toilet paper is associated with the perceived threat of COVID-19 (Garbe et al. 2020), our results also supported the role of threat perception. However, in the present study, we differentiated between the fear of contracting COVID-19 and the fear related to the severity of the disease. Moreover, we also assessed whether people felt threatened by a possible shortage of commodities. Among these threat perceptions, only the perceived severity of a COVID-19 infection was related to hoarding behavior. Most studies that found a substantial relationship between threat perceptions and stockpiling focused on the impact of perceived severity rather than perceived susceptibility to it, asking participants how much they felt threatened by COVID-19 (Garbe et al. 2020) or how serious/life-threatening they considered COVID-19 to be (Giroux et al. 2021; Kim et al. 2020). However, Nowak et al. (2020) distinguished between perceived severity and perceived susceptibility to COVID-19 and, in contrast to the current study, found a significant association of both dimensions of threat perception with stockpiling. Possible explanations for the different results could be cultural factors, as the samples were from different countries (i.e., Poland and Germany), general differences in the study population, as the Nowak et al. (2020) sample was more balanced in terms of gender and older on average, or the survey period, as our survey began 2 weeks later. In addition, differences in findings may be due to the use of different scales to measure susceptibility. Whereas Nowak et al. (2020) primarily assessed the perceived likelihood of developing COVID-19, our assessment of vulnerability was somewhat broader and included items on the likelihood of close relatives becoming ill and on the vulnerability to complications due to one's state of health. Therefore, further cross-national research that takes a differentiated look at threat perceptions is needed to understand the role of perceived severity and susceptibility in the context of stockpiling.

In line with research focusing on personality determinants of stockpiling, we found that these severity perceptions were related to emotionality (Garbe et al. 2020). Additionally, age and health concerns could explain variance in the perceived severity of COVID-19, suggesting the need to target effective communication to at-risk groups.

We found a significant effect of descriptive norms, suggesting that observation of actual behavior weighed more heavily for panic buying than did the perceived acceptance of the behavior, which could not significantly predict stockpiling behavior. This result is consistent with the literature on social norms, which finds a stronger relationship between descriptive norms and behavior than between injunctive norms and behavior (Manning 2009). In light of this result, it is possible that the continuous media reporting on panic buying at the outbreak of COVID-19 may have further fanned the flames of inappropriate stockpiling behavior.

As for the other variables in our proposed model, we found significant associations between perceived barriers and benefits and stockpiling behavior and a rather small effect of self-efficacy beliefs. Literature suggests a predictive superiority of benefits and barriers over threat perception (Carpenter 2010). While we also found perceived barriers to be the strongest predictor of stockpiling, we found a comparatively smaller but significant effect of perceived benefits. This is in contrast to Roşu et al. (2021) and Nowak et al. (2020), who found no significant effect of perceived benefits, which is most likely due to different conceptualizations of perceived benefits. While our study directly examined the benefits of stockpiling behavior (e.g., being prepared for all contingencies, feeling of security), Nowak et al. (2020) focused on the benefits of preventive measures (handwashing, following recommendations) and Roşu et al. (2021) captured personal and economic benefits of adjusting the consumption level to a sustainable level and thus measured benefits of reducing panic buying.

Interestingly, benefits and barriers were both mainly predicted by cognitive attitudes towards stockpiling, which suggests a conscious evaluation of the advantages and disadvantages, representing a cognition-driven component in our model compared to the highly emotional threat perceptions. Furthermore, we found that higher levels of conscientiousness were associated with a tendency to perceive stockpiling as beneficial. Since it has already been reported that more conscientious people are more likely to hoard toilet paper than less conscientious ones (Garbe et al. 2020), it might be plausible that particularly conscientious people perceive more advantages of stockpiling and are therefore more prone to over-purchase than less conscientious people. Additionally, we found that people who score high on honesty perceive stockpiling as less beneficial and report more barriers than people scoring low on honesty, reflecting previous results showing that high levels of honesty are associated with less stockpiling (Columbus 2021).

Finally, as the measurement model of stockpiling behavior shows, panic buying was characterized primarily by stockpiling of grain products, toilet paper, kitchen utensils, and canned goods. However, as the mean values and the standard deviations of the purchase ratings of the respective product groups show, we observed only a moderate increase in buying in comparison to what one might assume based on media reports, which often promote extreme individual cases. In the present sample, the mean value of total stockpiling was 4.13 (SD = 0.30), and for each product group subjects could indicate whether they had purchased very much less (1) to very much more (7) than would have been usual for their regular purchase. Therefore, we argue that the estimate of the prevalence of hoarding may be biased due to the availability of numerous examples of hoarding behavior in the media.

In sum, our results are in line with previous research, providing further support for the relevance of threat perceptions and the role of emotionality and honesty in particular. Furthermore, we extend these findings by showing that perceived barriers and benefits and descriptive social norms might be important variables for the explanation of hoarding.

## Limitations

The present study is subject to some limitations. First, we recruited a convenience sample which ultimately led to partly limited sample representativeness, especially regarding the gender ratio. Furthermore, young people and students tended to be overrepresented. Thus, our study sample is not representative for the German population, which might in turn affect the generalizability of our reported findings to the general population. In particular, older people who feel more at risk might differ in their threat perception and the relationship pattern of this perception with stockpiling attitudes. However, when compared to the study published by Nowak et al. (2020), whose sample was more representative, many of the results are consistent, particularly with respect to perceived barriers and perceived severity.

Second, despite our efforts to immediately react to the sudden increase in retail demand, our survey period started shortly after the peak in stockpiling, which is dated around the 12th calendar week (Statistisches Bundesamt 2020). However, as indicated by our measurement model, we were still able to successfully assess stockpiling behavior, since the significant indicators were products typically associated with panic buying.

## Conclusion

The present study contributes to a better understanding of stockpiling behavior through a comprehensive investigation of possible explanatory variables. Most importantly, we were able to show that perceived barriers and benefits of stockpiling were relevant predictors of panic buying. Our study also provided evidence that people who feel threatened by COVID-19 infection were more prone to build up stocks and engage in panic buying. Specifically, the perceived severity of infection emerged as the only significant predictor of stockpiling among COVID-19- and shortage-related threat perceptions. Interestingly, our data suggest that stockpiling behavior was not motivated by the fear of a potential shortage of essential commodities. Finally, we found that social cues derived from observing the shopping behavior of others are relevant for predicting stockpiling behavior. Based on these findings, we emphasize the relevance of differentiated assessment of perceived threat and propose targeted interventions aimed at reducing perceived benefits of stockpiling and severity beliefs related to COVID-19, as well as emphasizing disadvantages of stockpiling and reducing media exposure of stockpiling behavior to prevent panic buying.

## Figures and Tables

**Table 8:**
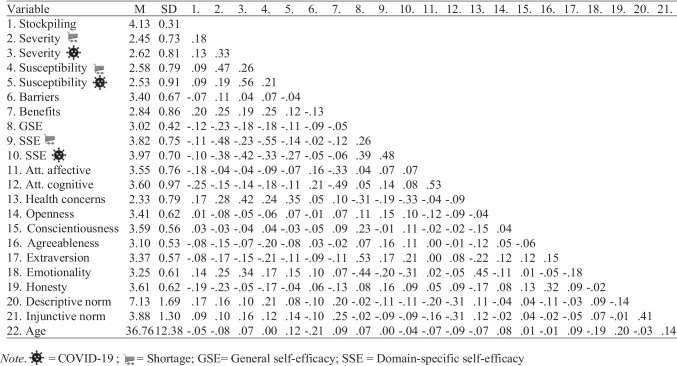
Bivariate correlations between all constructs of interest

**Fig. 3:**
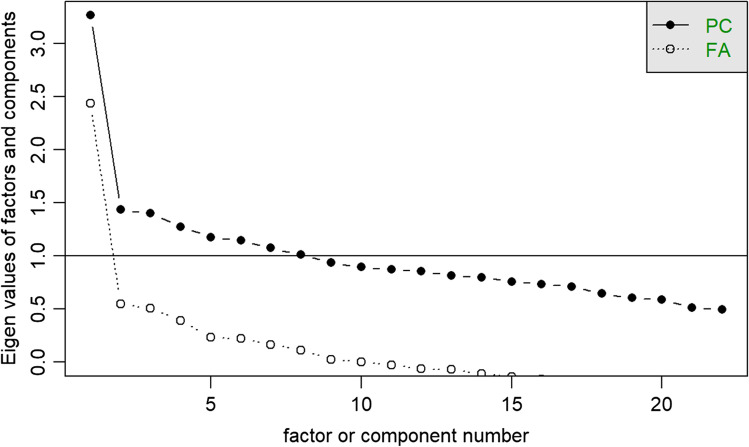
Screeplot of stockpiling scale

**Table 9:**
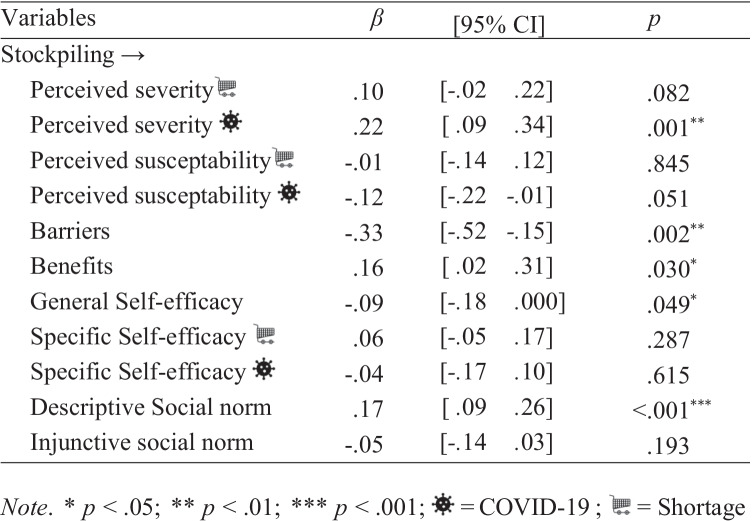
Predictors of stockpiling

**Table 10:**
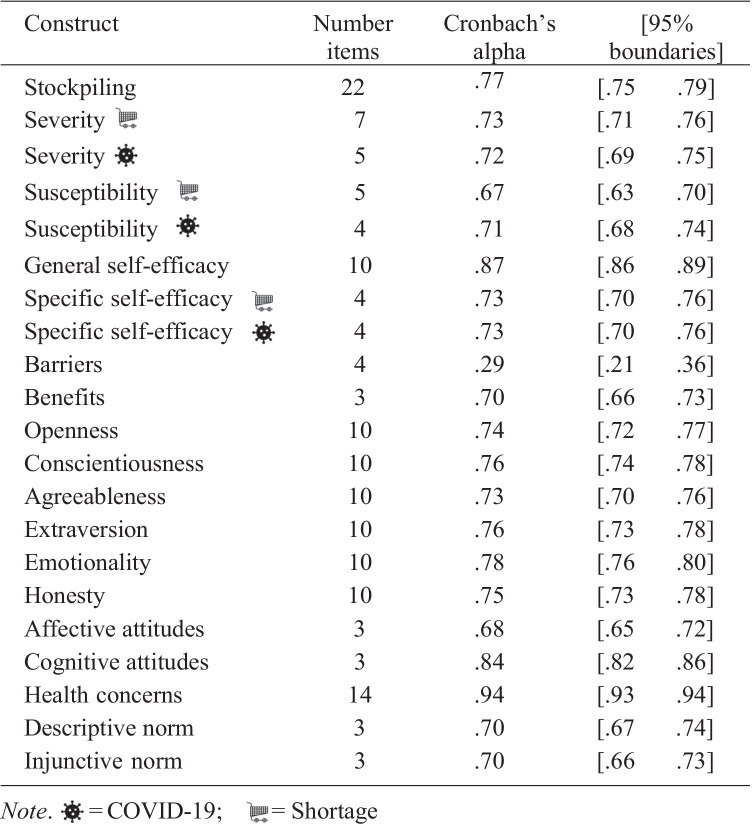
Cronbach’s alpha for all scales administered in the survey

## Data Availability

The study materials, dataset, and analysis script are available on the Open Science Framework (OSF; https://osf.io/xyct7/).

## Appendix B

### Screeplot of stockpiling variables

Fig. 3

## Appendix C

### Screeplot of stockpiling variables

Table 9

## Appendix D

### Internal consistency of administered scales

Table 10

## Footnotes

1 We thank an anonymous reviewer for pointing out that much of the research discussed in the present manuscript was conducted at the same time that the present study was conducted. This burst of research at the onset of the pandemic was not surprising, as the changes to everyday life caused many challenges related to public health but also individual health-related behavior. Hence, our study does not build upon these findings in the way that it was planned based on them; rather, we aim to incorporate related findings into the theoretical framework discussed in the present manuscript.
